# Efficacy and Safety of Rituximab in the Treatment of Vasculitic Leg Ulcers Associated with Hepatitis C Virus Infection

**DOI:** 10.1155/2012/923897

**Published:** 2012-12-16

**Authors:** Fabio Bonilla-Abadía, Andrés F. Echeverri, Jorge H. Izquierdo, Felipe Cañas, Carlos A. Cañas

**Affiliations:** ^1^Rheumatology Unit, Fundación Valle del Lili, ICESI University, Cali, Colombia; ^2^Internal Medicine Department, Fundación Valle del Lili, ICES University, Cali, Colombia; ^3^Fundación Valle del Lili and Medical School, University of Valle, Cali, Colombia

## Abstract

Vasculitic leg ulcers are a cutaneous manifestation of hepatitis C virus (HCV) infection often associated with cryoglobulinemia. Their treatment is difficult and is based on steroids and immunosuppressive drugs with an erratic response and a high probability of adverse reaction. We report three patients with vasculitic leg ulcers associated with hepatitis C virus infection who were treated successfully with rituximab. The pain control and healing were achieved quickly. No adverse effects with rituximab in these patients were presented.

## 1. Introduction

Infection with hepatitis C virus (HCV) causes chronic liver disease in approximately 80% of cases, resulting in chronic inflammation and cirrhosis [1]. It is frequently associated with autoimmune extrahepatic complications including systemic vasculitis [2]. Its degree of compromise is variable, ranging from mild to fulminant often affecting nerves and kidney. Immune complex mediated is the most common form of vasculitis in HCV infection, which involves the small vessels (venules, capillaries, and arterioles) and is associated or not to cryoglobulinemia [3]. Skin is one of the most frequently affected organs [4]. Clinical characteristics comprise palpable purpura and very painful leg ulcers. These ulcers tend to be chronic and difficult to treat with a significant impact on psychosocial aspects as well as on physical well-being [5].

Bearing in mind recent reports of the effectiveness and safety of rituximab in cases of cryoglobulinemic vasculitis associated with HCV infection [6], we decided to treat three patients with vasculitis leg ulcers associated to chronic HCV infection refractory to steroids and with contraindications to receive cytotoxic treatment. The patients achieved a rapid control of pain and scarring.

## 2. Methods

Between August 2001 and September 2010, we treated 16 patients with autoimmune phenomena triggered by HCV infection in a tertiary health care center in Cali, Colombia. Three patients with steroids refractory vasculitic leg ulcers were treated with rituximab.

### 2.1. Case 1

A 73-year-old woman was admitted because painless ulcers on the lower limbs which appeared six months earlier. Her past medical history was remarkable for a long-lasting progressive demyelinating disease of spinal cord.

On examination, extensive palpable purpura and ulcers on the lower limbs were observed. Her cardiopulmonary examination was normal. Alteration of proprioception and ataxia were evidenced. Laboratory exams showed elevated sedimentation rate 58 mm/h, C3 54.7 mg/dL (90–180), C4 11.8 mg/dL (10–40), and a positive direct Coombs test. Complete blood count, clotting studies, bilirubin level, and urine analysis were normal, as well as aspartate aminotransferase (AST) and alanine aminotransferase (ALT) levels. Quantitative test for antibodies to hepatitis C virus in serum (anti-HCV-ELISA) was 113 (<0.1) with high viral load, and HCV genotyping showed Ib genotype. Hepatitis B surface antigen and VIH test were negative. Autoimmunity tests revealed positivity for antinuclear antibodies (ANAs) 1/320, rheumatoid factor 40 (<14), anti-Ro 66 U (<20), and anti-La 151 U (<20). Schirmer test and lip biopsy were normal. The biopsy of the skin lesion on the leg revealed leukocytoclastic vasculitis. Cryoglobulins were not detectable.

The diagnosis of HCV infection with severe cutaneous vasculitis was done. She was treated for two weeks with 1 mg/Kg of prednisone unsuccessfully. Then, we started rituximab (initial dose of 1 g, and a second dose of 1 g was given 2 weeks later). Her clinical condition related to fatigue, palpable purpura and leg ulcers showed rapid and significant improvement. Steroid dose was slowly tapered. Neurological status remained unchanged.

### 2.2. Case 2

A 73-year-old woman was admitted because of quickly progressive painful ulcers and purpuric lesions on the lower limbs which appeared ten months earlier. Other complaints were xerostomia and polyarthralgia. A recent diagnosis of HCV infection was made based on anti-HCV-ELISA and viral load of 1.890.000 copies with HCV Ib genotype. Her medical history included arterial hypertension and diabetes mellitus.

**Figure 1:**
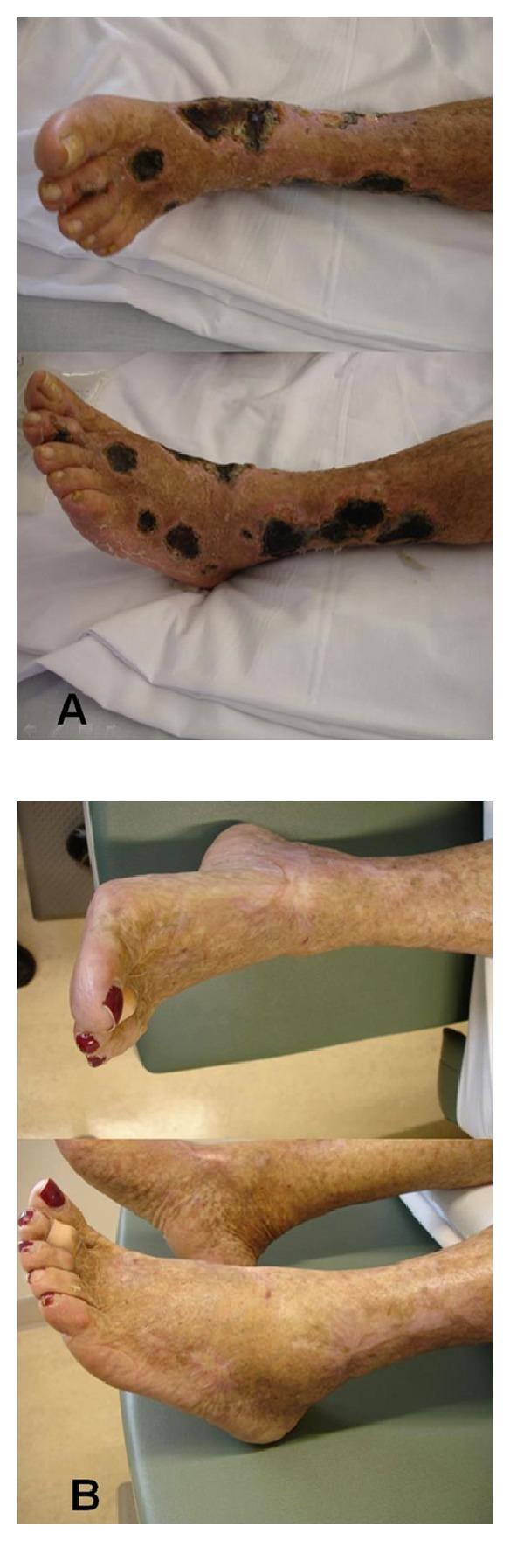
Leg ulcers associated to HCV infection in the case 2. (a) Pre- and (b) post-treatment with rituximab.

At physical examination on admission, we observed xerostomia without parotid swelling or oral ulcers. Cardiopulmonary, digestive, neurological, and osteoarticular systems were normal. In the lowers limbs, palpable purpuric lesions were presented as well as deep, painful and extensive vasculitic necrotic ulcers up to 4 cm (Figure 1(a)). She also had a low C4 (8 mg/dL) and C3 (102 mg/dL) and sedimentation rate at 89 mm/h. Hepatitis B surface antigen and anti-HBs antibodies were negative. Autoimmunity tests revealed a rheumatoid factor of 106; ANAs, ENAs and direct Coombs test were negative. Cryoglobulins were not detectable. The biopsy of the skin lesion on the right leg-revealed leukocytoclastic vasculitis. A diagnosis of HCV, associated systemic vasculitis with several cutaneous necrotic leg ulcers was made, and initial management with steroid (1 mgr/Kg of prednisone), opioid, and local treatment was established. Because no adequate response was observed, rituximab was started (initial dose of 1 g, and a second dose of 1 g was given 2 weeks later). Subsequent consultation showed progressive improvement in clinical condition and general symptoms, and at three months the ulcers resolved completely (Figure 1(b)).

### 2.3. Case 3

A 68-year-old woman was admitted because chronic multiple ulcerated lesions in legs with skin biopsy reported leukocytoclastic vasculitis. The past medical history include arterial hypertension, hypothyroidism secondary to thyroidectomy for benign nodules, and a long-lasting (12 years) HCV infection secondary to blood transfusion and treated with interferon and ribavirin. She had also polyarthralgias and dysesthesias in legs.

On admission, physical examination revealed hyperpigmented macules with livedo reticularis, numerous painful leg ulcers. Cardiopulmonary and gastrointestinal examination were normal.

Laboratory findings included erythrocyte sedimentation rate at 60 mm/h, leukocyte count of 3370 cells/mm^3^ with normal differential count; platelet count, hemoglobin, and urine analysis were normal too. Low C3 (65 mg/dL) and C4 (11 mg/dL) increased ALT 141 U/L and AST 160 U/L with normal bilirubin level. Rheumatologic test showed a rheumatoid factor of 69 (<14); ANAs 1/40, ANCAs, and ENAS were negative. Serologic studies revealed positive HCV antibodies and elevated viral count at 900.000 copies. Cryoglobulins were not detectable. Diagnosis of HCV associated-systemic vasculitis with severe leg ulcers is made. The patient was treated with intravenous hydrocortisone. Knowing the severity of the condition and refractory to previous treatment, interferon, ribavirin, and rituximab regimens were added; the latter with 2 doses of 1 gr separated two weeks. A rapid control of pain, healing of leg ulcers with reduction of steroid, and analgesics needs were achieved.

## 3. Discussion

The leg ulcers secondary to cutaneous vasculitis are a form of extrahepatic manifestation of HCV infection, which may or may not be associated to cryoglobulins [7]. We show three female patients with HCV infection and severe vasculitis leg ulcers refractory to initial treatment with steroids. They had contraindication of aggressive immunosuppressive drugs.

Rituximab treatment was initiated according to the effectiveness and safety reported elsewhere [6]. The association of mixed cryoglobulinemia with HCV infection and systemic vasculitis may be linked to the ability of the HCV to infect B cells [8] and induce their clonal expansion that may lead to the production of organ-specific autoantibodies [9]. Rituximab, a chimeric monoclonal antibody that binds to the B-cell surface antigen CD20, interferes with monoclonal IgM production, cryoglobulin synthesis, and organ deposition of immune complexes and has shown to be highly effective in modifying the dynamics of B cells by deleting expanded clones and markedly improving of mixed cryoglobulinemia syndrome safely [10]. The treatment outcomes with rituximab of systemic vasculitis in patients with hepatitis C virus infection with and without detectable mixed cryoglobulinemia have been reported as similar, although in small numbers of patients [7].

The three patients shown in this work had evident cutaneous vasculitis with leg ulcers without cryoglobulins or other relevant organic involvements. They were not responders to steroids and had some contraindications to receive cytotoxic treatment. Rituximab was given at an initial dose of 1 gr. and at a second dose of 1 gr. two weeks later. This treatment induced a favorable clinical response with rapid pain control and resolution of the skin lesions as also improved psychosocial functioning, which allowed better adherence to continuity of care in the three patients. The treatment permitted the minimal steroid use. No adverse effects related to rituximab treatment were observed.

Therefore, rituximab is an effective and a safe treatment for severe vasculitis leg ulcers associated with HCV infection.
